# The Infectivity Gene *bbk13* Is Important for Multiple Phases of the Borrelia burgdorferi Enzootic Cycle

**DOI:** 10.1128/IAI.00216-21

**Published:** 2021-09-16

**Authors:** George F. Aranjuez, Amanda G. Lasseter, Mollie W. Jewett

**Affiliations:** a Division of Immunity and Pathogenesis, Burnett School of Biomedical Sciences, University of Central Florida College of Medicine, Orlando, Florida, USA; UC Davis School of Veterinary Medicine

**Keywords:** *Borrelia burgdorferi*, Lyme disease, *bbk13*, host-pathogen interactions, immune evasion, tick transmission, vector-borne diseases

## Abstract

Lyme disease is a multistage inflammatory disease caused by the spirochete Borrelia burgdorferi transmitted through the bite of an infected Ixodes scapularis tick. We previously discovered a B. burgdorferi infectivity gene, *bbk13*, that facilitates mammalian infection by promoting spirochete population expansion in the skin inoculation site. Initial characterization of *bbk13* was carried out using an intradermal needle inoculation model of mouse infection, which does not capture the complex interplay of the pathogen-vector-host triad of natural transmission. Here, we aimed to understand the role of *bbk13* in the enzootic cycle of B. burgdorferi. B. burgdorferi spirochetes lacking *bbk13* were unable to be acquired by naive larvae fed on needle-inoculated mice. Using a capsule feeding approach to restrict tick feeding activity to a defined skin site, we determined that delivery by tick bite alleviated the population expansion defect in the skin observed after needle inoculation of Δ*bbk13* B. burgdorferi. Despite overcoming the early barrier in the skin, Δ*bbk13* B. burgdorferi remained attenuated for distal tissue colonization after tick transmission. Disseminated infection by Δ*bbk13* B. burgdorferi was improved in needle-inoculated immunocompromised mice. Together, we established that *bbk13* is crucial to the maintenance of B. burgdorferi in the enzootic cycle and that *bbk13* is necessary beyond early infection in the skin, likely contributing to host immune evasion. Moreover, our data highlight the critical interplay between the pathogen, vector, and host as well as the distinct molecular genetic requirements for B. burgdorferi to survive at the pathogen-vector-host interface and achieve productive disseminated infection.

## INTRODUCTION

Lyme disease remains the predominant vector-borne disease in the United States (1). Borrelia burgdorferi, the causative agent of Lyme disease (2, 3), is transmitted to humans via the bite of an infected Ixodes scapularis tick (4). The acute manifestations of Lyme disease involve fever, nausea, and, in some cases, a characteristic bull’s-eye rash (erythema migrans) at the tick bite site. If undiagnosed and untreated, Lyme disease progresses to a late stage as B. burgdorferi disseminates and colonizes various distal tissues such as the joints (Lyme arthritis), the heart (Lyme carditis), and the nervous system (neuroborreliosis) (5).

In nature, the B. burgdorferi reservoir is maintained by the enzootic cycle of transmission and acquisition of B. burgdorferi from various vertebrate hosts as the Ixodes scapularis tick feeds and progresses through its life cycle (6). After hatching, an unfed *Ixodes* larva will feed on blood from small vertebrate hosts such as rodents, triggering it to molt to the nymph stage. If the host is infected, the larva can acquire B. burgdorferi with the blood meal. B. burgdorferi will then proliferate in the fed larva and persist through the molting phase, which then gives rise to infected unfed nymphs. It is primarily the nymph stage of *Ixodes* that transmits B. burgdorferi to humans. The risk of contracting Lyme disease rises in areas with high numbers of B. burgdorferi-infected nymphal ticks (7). Nymphs then molt into sexually mature adults, which mate and lay eggs to give rise to the next generation of uninfected *Ixodes* ticks.

The molecular mechanisms that promote B. burgdorferi infection of its mammalian host are not completely understood, and this has slowed the development of new strategies to curb the rising prevalence of Lyme disease. Genetic manipulation of B. burgdorferi coupled with a mouse model of infection has been central to the discovery of novel B. burgdorferi infectivity genes. Needle inoculation of mouse models of infection allows the precise control of both the inoculum dose and route of inoculation, enabling detailed characterization of infection kinetics. However, needle inoculation is an artificial route of infection that excludes the contribution of the tick vector to B. burgdorferi transmission—both its influence on B. burgdorferi itself and its influence on the host microenvironment at the site of feeding. B. burgdorferi adapts to the conditions present in the unfed tick and undergoes significant transcriptional changes during tick feeding, potentiating its infectivity (8, 9). Moreover, the feeding activity of the ticks themselves, particularly the influence of the tick saliva on the skin feeding site, affects B. burgdorferi infection (10–12). The tick feeding site is unique in that it is the combined interaction of the pathogen, the vector, and the mammalian host, which is likely critical to the adaptation and mechanisms of pathogenesis of B. burgdorferi as well as other tick-borne pathogens.

We recently characterized the role of the novel B. burgdorferi infectivity gene *bbk13* in the early phase of B. burgdorferi mammalian infection. Using an intradermal needle inoculation model of infection, we showed that *bbk13* is important for spirochete population expansion in the skin, which then drives the downstream steps of disseminated infection, culminating in B. burgdorferi colonization of distal tissues (13). Consequently, the loss of *bbk13* leads to reduced colonization of distal tissue sites such as the skin, heart, and joints (13). Furthermore, we have established that BBK13 forms large oligomers in the spirochete membrane and is highly immunogenic (13, 14). In this work, we used *Ixodes* ticks and mice to study the role of *bbk13* in the natural enzootic cycle of B. burgdorferi. We demonstrate that *bbk13* is similarly required for productive disseminated infection when delivered by tick bite transmission. Interestingly, B. burgdorferi inoculation by tick feeding bypassed the early requirement for *bbk13* to promote B. burgdorferi population expansion in the skin. Despite this, *bbk13* remained critical for efficient colonization of distal tissues. In addition, we show that distal tissue colonization by Δ*bbk13* B. burgdorferi is restored in an immunocompromised mouse host. This work points toward a role of *bbk13* in host immune evasion and uncovers a more complex model of *bbk13* function in B. burgdorferi mammalian infection, emphasizing the impact of the natural tick vector on the establishment of B. burgdorferi infection as well as the distinct molecular genetic requirements for B. burgdorferi survival during early localized infection at the pathogen-vector-host interface and the establishment of productive disseminated infection of distal host tissues.

## RESULTS

### Naive larvae fail to acquire Δ*bbk13* B. burgdorferi from infected mice.

**FIG 1:**
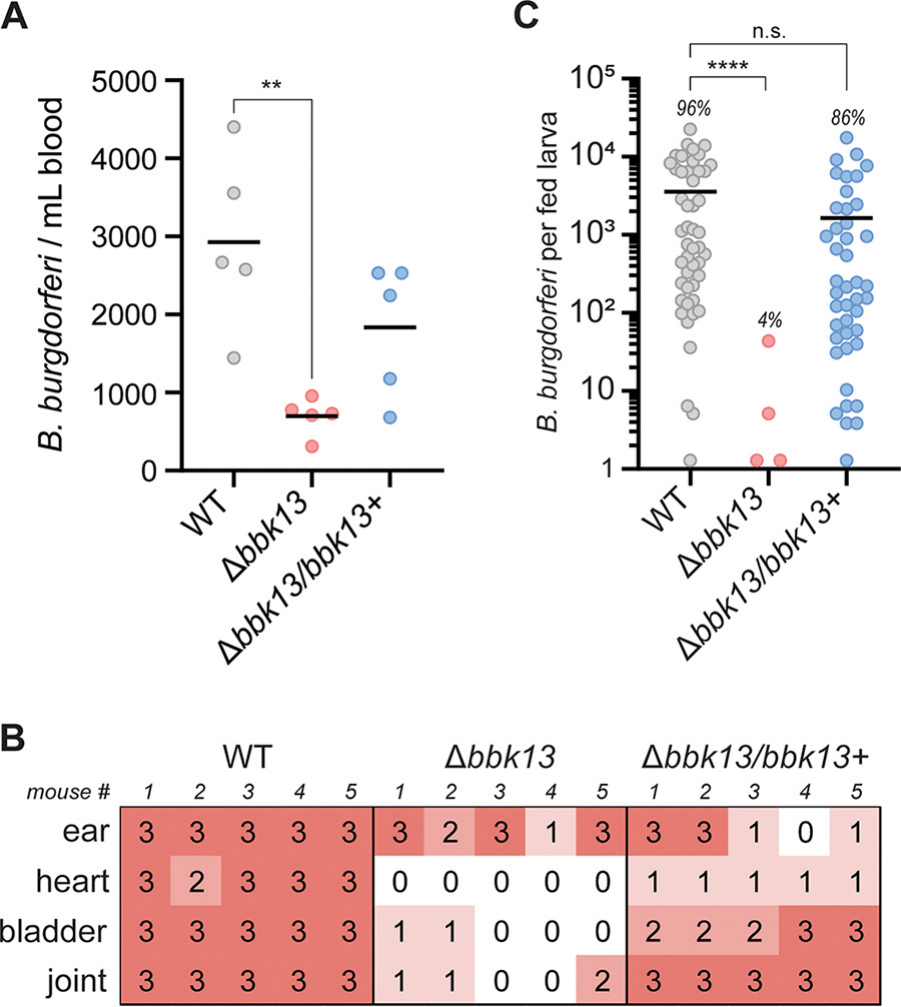
Naive larval ticks fail to acquire Δ*bbk13* B. burgdorferi during feeding on infected mice. Groups of six C3H/HeN mice were needle inoculated intradermally with 10^4^ WT, Δ*bbk13*, or Δ*bbk13*/*bbk13*^+^ B. burgdorferi spirochetes. (A) At 6 days postinoculation, disseminating B. burgdorferi spirochetes were enumerated by plating blood in solid BSK-agarose medium and enumerating CFU. Each dot represents an individual mouse. The mean value is indicated by a black horizontal line. The Kruskal-Wallis test with Dunn’s multiple comparisons was used to determine statistical significance (**, *P* ≤ 0.01). (B) At 4 weeks postinoculation, the indicated tissues were assessed for B. burgdorferi by a reisolation assay. Semiquantitative scoring of reisolation cultures was performed (“0” indicates no spirochetes, and “1” to “3” indicate increasing spirochete densities). (C) Naive tick larvae fed to repletion on C3H/HeN mice 3 weeks after needle inoculation with 10^4^ WT, Δ*bbk13*, or Δ*bbk13*/*bbk13*^+^ B. burgdorferi spirochetes. Individual fed larvae were homogenized and plated in solid BSK-agarose medium, and B. burgdorferi loads were determined by enumerating CFU. Each data point represents an individual tick. The tick infection rate for each B. burgdorferi clone is indicated above the corresponding scatterplot. The mean is indicated by a horizontal black line. The Kruskal-Wallis test with Dunn’s multiple comparisons was used to determine statistical significance (****, *P* < 0.0001; n.s., not significant).

B. burgdorferi’s enzootic cycle depends not only on successful tick transmission but also on its acquisition from the animal host by naive larvae. Needle inoculation of Δ*bbk13* B. burgdorferi into mice leads to reduced, but not absent, colonization of distal tissues, including the skin (13). This allowed us to ask whether Δ*bbk13* B. burgdorferi can be acquired from infected mice by feeding *Ixodes* larvae. We needle inoculated cohorts of C3H/HeN mice with 10^4^ wild-type (WT), Δ*bbk13*, or Δ*bbk13*/*bbk13*^+^ B. burgdorferi spirochetes intradermally to generate infected mice for naive tick feeding. Consistent with what we have shown previously (13), Δ*bbk13* B. burgdorferi spirochetes were found at reduced levels during blood dissemination (Fig. 1A) and in distal tissues (Fig. 1B). More importantly, Δ*bbk13* B. burgdorferi was still present in the skin, as indicated by reisolation from the ear (Fig. 1B, top row). B. burgdorferi colonization of tissues was assessed via semiquantitative tissue reisolation assay (see Materials and Methods). Briefly, reisolation cultures were inspected daily for the presence of spirochetes by dark-field microscopy. A numerical score was assigned to each culture corresponding to the observed density of spirochetes (0 for no spirochetes and 1 to 3 depending on the number of spirochetes).

At 3 weeks postinoculation, naive *Ixodes* larvae were fed to repletion on the three groups of B. burgdorferi-infected mice. Individual fed larvae were assessed for B. burgdorferi acquisition via homogenization and plating in solid Barbour-Stoenner-Kelly (BSK)-agarose medium to quantify the number of B. burgdorferi spirochetes per tick. The rate of acquisition of wild-type B. burgdorferi after larval tick feeding was 96% (48/50 larvae), with an average of ∼5 × 10^3^ B. burgdorferi spirochetes per tick (Fig. 1C). In stark contrast, only 4% of the *Ixodes* larvae (4/100 larvae) that fed on Δ*bbk13* B. burgdorferi-infected mice acquired spirochetes and with B. burgdorferi loads at dramatically reduced levels (Fig. 1C). The rate of acquisition of Δ*bbk13*/*bbk13*^+^ B. burgdorferi from infected mice was 86% (43/50 larvae), with the average number of B. burgdorferi spirochetes per tick being comparable to that of the wild-type B. burgdorferi group (Fig. 1C). These data indicated that there is a significant deficiency in the ability of *Ixodes* larvae to acquire spirochetes from Δ*bbk13* B. burgdorferi-infected mice.

### Increasing the inoculum dose does not rescue low Δ*bbk13* B. burgdorferi numbers in distal tissues.

**FIG 2:**
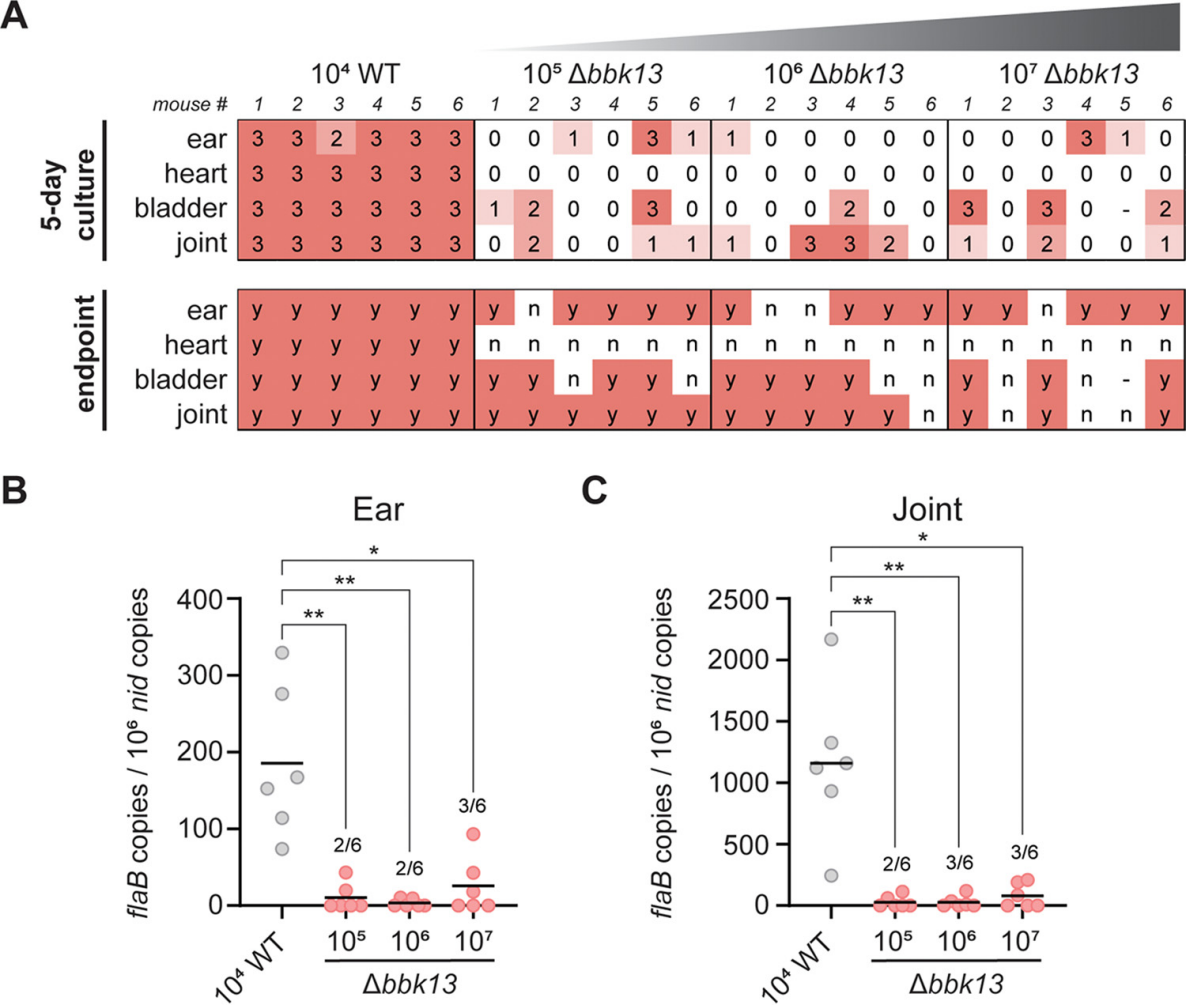
Increasing the inoculum dose does not alleviate the infectivity defect of Δ*bbk13* B. burgdorferi. Groups of six C3H/HeN mice were intradermally inoculated with 10^4^ WT B. burgdorferi or increasing doses (10^5^, 10^6^, and 10^7^) of Δ*bbk13* B. burgdorferi spirochetes. (A) At 3 weeks postinoculation, tissues were assessed for B. burgdorferi by a reisolation assay. Semiquantitative scoring of reisolation cultures was performed following 5 days (0 indicates no spirochetes, and 1 to 3 indicate increasing spirochete densities) and 14 days (endpoint, scored for the presence [“y”] or absence [“n”] of spirochetes) of culture incubation. (B and C) Total DNA was extracted from the ears (B) and joints (C). The B. burgdorferi load was measured by quantifying B. burgdorferi *flaB* copies normalized to 10^6^ mouse *nid* copies using quantitative PCR. In cases where not all samples per group had detectable B. burgdorferi DNA, the numbers of positive samples out of the total mice per group are indicated in the scatterplot. Data points below the level of detection are not shown. The mean is indicated by a horizontal black line. The Kruskal-Wallis test with Dunn’s multiple comparisons was used to determine statistical significance. (*, *P* < 0.05; **, *P* < 0.01).

For *Ixodes* ticks to acquire B. burgdorferi through feeding, the spirochetes must be present in sufficient numbers at the skin feeding site. Although both wild-type and Δ*bbk13* B. burgdorferi spirochetes were present at a skin site (Fig. 1B, ear), Δ*bbk13* B. burgdorferi spirochetes were found at reduced numbers compared to wild-type B. burgdorferi during late infection (13). Reduced Δ*bbk13* B. burgdorferi numbers in the skin likely negatively influence acquisition via tick feeding, raising the question of whether low Δ*bbk13* B. burgdorferi acquisition was due to reduced spirochete numbers and/or a molecular function of BBK13. To address this, we attempted to increase the Δ*bbk13* B. burgdorferi load in the skin by increasing the inoculum dose. We needle inoculated 10^5^, 10^6^, or 10^7^ Δ*bbk13* B. burgdorferi spirochetes intradermally into C3H/HeN mice. At 3 weeks postinoculation, distal tissues were collected and assessed for B. burgdorferi colonization via the semiquantitative tissue reisolation assay (13). After 5 days of incubation, nearly all of the reisolation cultures from mice inoculated with 10^4^ wild-type B. burgdorferi spirochetes demonstrated a score of “3” (23/24 cultures) (Fig. 2A). At the same time point, reisolation cultures from mice inoculated with increasing doses of Δ*bbk13* B. burgdorferi were mostly negative for spirochetes (10^5^ dose, 9/24 positive; 10^6^ dose, 6/24 positive; 10^7^ dose, 8/24 positive), and the positive cultures demonstrated varying spirochete densities (scores ranging from 1 to 3) (Fig. 2A). Thus, no observable increase in distal tissue colonization was detected upon increasing the inoculum dose of Δ*bbk13* B. burgdorferi. These trends were maintained at the 2-week endpoint of the reisolation assay (Fig. 2A). Direct measurement of B. burgdorferi loads in the distal tissues by quantitative PCR (qPCR) supported the findings in the tissue reisolation assay. Despite the increased inoculum doses, the mean Δ*bbk13* B. burgdorferi loads in the ears and joints remained consistently lower than those of wild-type B. burgdorferi at a lower inoculum (Fig. 2B and C). Moreover, Δ*bbk13* B. burgdorferi loads were above the level of detection in only 2 or 3 tissues out of 6 mice per inoculum group, further emphasizing the reduced efficiency of distal tissue colonization of Δ*bbk13* B. burgdorferi.

### *bbk13* is dispensable for B. burgdorferi maintenance in *Ixodes* ticks.

**FIG 3:**
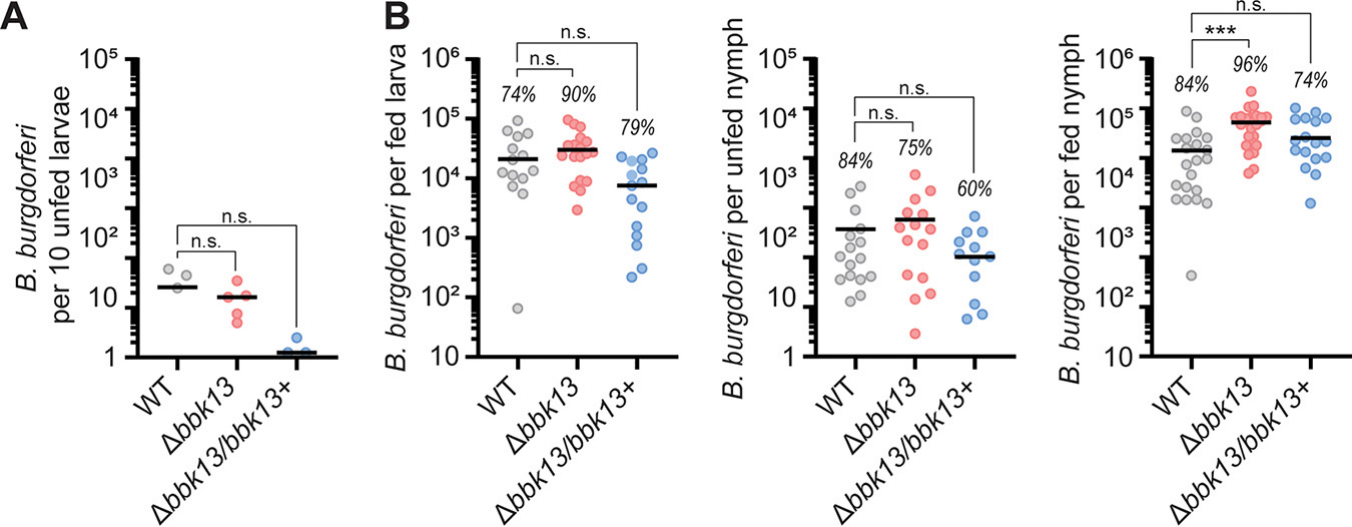
*bbk13* is dispensable for replication and survival in ticks. Naive larvae were artificially infected by immersion with wild-type (WT), Δ*bbk13*, or Δ*bbk13/bbk13*^+^ B. burgdorferi. B. burgdorferi loads in ticks at various stages of development were measured by plating tick homogenates in solid BSK-agarose medium and enumerating CFU. (A) Unfed larvae were assessed for B. burgdorferi loads following artificial infection. Each data point represents a group of 10 larvae. (B) B. burgdorferi loads in fed larvae, unfed nymphs, and fed nymphs were determined. Each data point represents an individual tick. The tick infection rate for each B. burgdorferi clone is indicated above the corresponding scatterplot. The mean is indicated by a horizontal black line. The Kruskal-Wallis test with Dunn’s multiple comparisons was used to determine statistical significance (***, *P* < 0.001; n.s., not significant).

To further understand the role of *bbk13* in the enzootic cycle of B. burgdorferi, we investigated the survival of Δ*bbk13* B. burgdorferi in ticks after the blood meal and through the molt. Given the inefficiency of Δ*bbk13* B. burgdorferi to be acquired by feeding ticks, we artificially infected unfed *Ixodes* larvae with wild-type, Δ*bbk13*, or Δ*bbk13*/*bbk13*^+^ B. burgdorferi by immersion (15). After recovery from immersion, positive infection was confirmed and quantified by plating homogenates from groups of 10 larvae per infecting B. burgdorferi clone. No significant difference was detected between the numbers of B. burgdorferi spirochetes per 10 larvae across the three B. burgdorferi clones (Fig. 3A). The infected larvae were fed to repletion on naive C3H/HeN mice. Approximately 7 days after feeding, 20 fed larvae per infecting B. burgdorferi clone were individually homogenized and plated in solid BSK-agarose medium to measure spirochete loads. Robust multilog expansion was detected for all B. burgdorferi clones (Fig. 3B, left), with no statistical difference between the average numbers of Δ*bbk13* or Δ*bbk13*/*bbk13*^+^ B. burgdorferi spirochetes and those of wild-type B. burgdorferi (Fig. 3B, left). A portion of the fed larvae were allowed to molt to unfed nymphs, from which 20 nymphs per B. burgdorferi clone were then individually assayed for B. burgdorferi loads by homogenization and plating for CFU. B. burgdorferi loads were comparable across all three groups and displayed the expected decline in number following the molt (Fig. 3B, middle) (16). The nymphs were then allowed to feed to repletion on mice, and the number of B. burgdorferi spirochetes per fed nymph was determined. No *bbk13*-dependent decrease in the B. burgdorferi load per fed nymph was detected; rather, the number of Δ*bbk13* B. burgdorferi spirochetes per fed nymph was found to be significantly increased compared to that of wild-type B. burgdorferi (Fig. 3B, right). Although not statistically significant, the average number of Δ*bbk13*/*bbk13*^+^ B. burgdorferi spirochetes in all tick stages except for fed nymphs tended to be lower than those of wild-type and Δ*bbk13* B. burgdorferi (Fig. 3), perhaps due to dysregulated expression of *bbk13* from the shuttle vector (13). In all, the loss of *bbk13* did not result in a defect in the replication and survival of B. burgdorferi in ticks.

### *bbk13* is critical for B. burgdorferi infection of mice by *Ixodes* nymph transmission.

**FIG 4:**
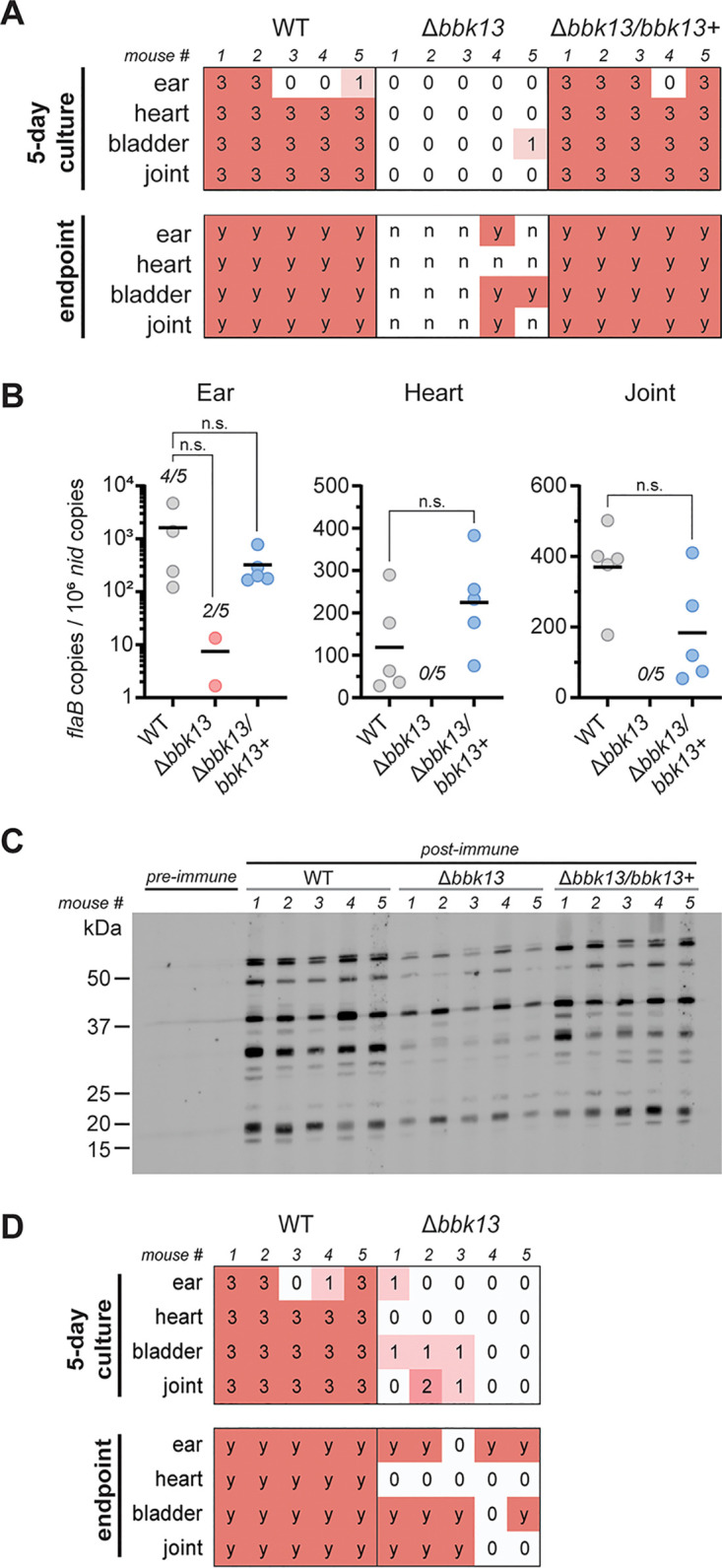
*bbk13* is critical for B. burgdorferi infection of mice by tick transmission. Unfed nymphs infected with wild-type (WT), Δ*bbk13*, or Δ*bbk13/bbk13*^+^ B. burgdorferi were fed to repletion on groups of five C3H/HeN mice. Mice were assessed for B. burgdorferi infection 3 weeks after tick feeding by a reisolation assay, quantitative PCR, and serology. (A) Distal tissues were collected for spirochete reisolation. Semiquantitative scoring of reisolation cultures was performed following 5 days (0 indicates no spirochetes, and 1 to 3 indicate increasing spirochete densities) and 14 days (endpoint, scored for the presence [y] or absence [n] of spirochetes) of culture incubation. (B) Total DNA was extracted from ear, heart, and joint tissues. B. burgdorferi loads in each tissue were measured by quantifying B. burgdorferi *flaB* copies normalized to 10^6^ mouse *nid* copies using quantitative PCR. The mean for each group is represented by a horizontal line. In cases where not all samples per group had detectable levels of B. burgdorferi DNA, the numbers of positive samples out of 5 mice per group are indicated. The Kruskal-Wallis test with Dunn’s multiple comparisons was used to determine statistical significance (n.s., not significant). (C) Preimmune and postimmune sera were assessed by immunoblotting for the presence of anti-B. burgdorferi antibodies. Post-immune serum from individual mice was used to blot total B. burgdorferi lysates. Preimmune sera were pooled across each of the three groups. Molecular weight standards are shown in kilodaltons. (D) Unfed *Ixodes* larvae were artificially infected with WT or Δ*bbk13* B. burgdorferi by immersion. Artificially infected larvae were allowed to feed freely to repletion on groups of five C3H/HeN mice. Three weeks after larva application, tissues were assessed for B. burgdorferi by a reisolation assay. Semiquantitative scoring of reisolation cultures was performed following 5 days (0 indicates no spirochetes, and 1 to 3 indicate increasing spirochete densities) and 14 days (endpoint, scored for the presence [y] or absence [n] of spirochetes) of culture incubation.

Our previous study used needle inoculation to demonstrate the importance of *bbk13* during mouse infection (13). In nature, B. burgdorferi is transmitted via the feeding activity of *Ixodes* ticks. Moreover, tick feeding influences both B. burgdorferi and the host to promote infection (6, 17). Unfed nymphs carrying equivalent loads of wild-type, Δ*bbk13*, or Δ*bbk13*/*bbk13*^+^ B. burgdorferi (Fig. 3B, middle) were used to test the requirement for *bbk13* during natural B. burgdorferi infection of mice by tick feeding. Three weeks after free feeding of infected nymphs on mice, distal tissues were collected and tested for B. burgdorferi colonization via the semiquantitative tissue reisolation assay. After 5 days of incubation, a majority (17/20) of the cultures containing tissues from mice fed on by wild-type B. burgdorferi-infected nymphs had a reisolation score of 3 (Fig. 4A, 5-day culture). In contrast, nearly all (19/20) reisolation cultures from mice fed on by Δ*bbk13* B. burgdorferi-infected nymphs had no spirochetes upon microscopic inspection and were given a score of 0 (Fig. 4A, 5-day culture). Moreover, at the 2-week endpoint, only 4 out of 20 Δ*bbk13* B. burgdorferi reisolation cultures were positive for spirochetes, with three of the five mice having no detectable spirochetes in any distal tissue examined (Fig. 4A, endpoint). In contrast, all reisolation cultures from the wild-type and Δ*bbk13*/*bbk13*^+^ B. burgdorferi-infected mouse tissues were positive for spirochetes at the 2-week endpoint (Fig. 4A, endpoint). Consistent with the observed deficit in tissue reisolation, Δ*bbk13* B. burgdorferi was below the level of detection by qPCR in the hearts (0/5 mice) and joints (0/5 mice) (Fig. 4B) of mice after tick feeding. Spirochete numbers were measurable in two out of five ear tissues from Δ*bbk13* B. burgdorferi-infected mice but were reduced compared to those in wild-type and Δ*bbk13*/*bbk13*^+^ B. burgdorferi-infected mice (Fig. 4B). Moreover, in agreement with reduced disseminating spirochetes and reduced overall loads in distal tissues, mice fed on by Δ*bbk13* B. burgdorferi-infected ticks exhibited a serological response with a reduced signal intensity yet a banding pattern similar to that of wild-type B. burgdorferi- or Δ*bbk13*/*bbk13*^+^ B. burgdorferi-infected mice (Fig. 4C), as demonstrated previously for mice infected with spirochetes lacking *bbk13* (13). The attenuated infection phenotype of Δ*bbk13* B. burgdorferi by nymph transmission was recapitulated by free-feeding artificially infected larvae (Fig. 4D), together supporting the importance of *bbk13* for B. burgdorferi infection by tick bite transmission.

### B. burgdorferi population expansion occurs at the tick feeding site.

**FIG 5:**
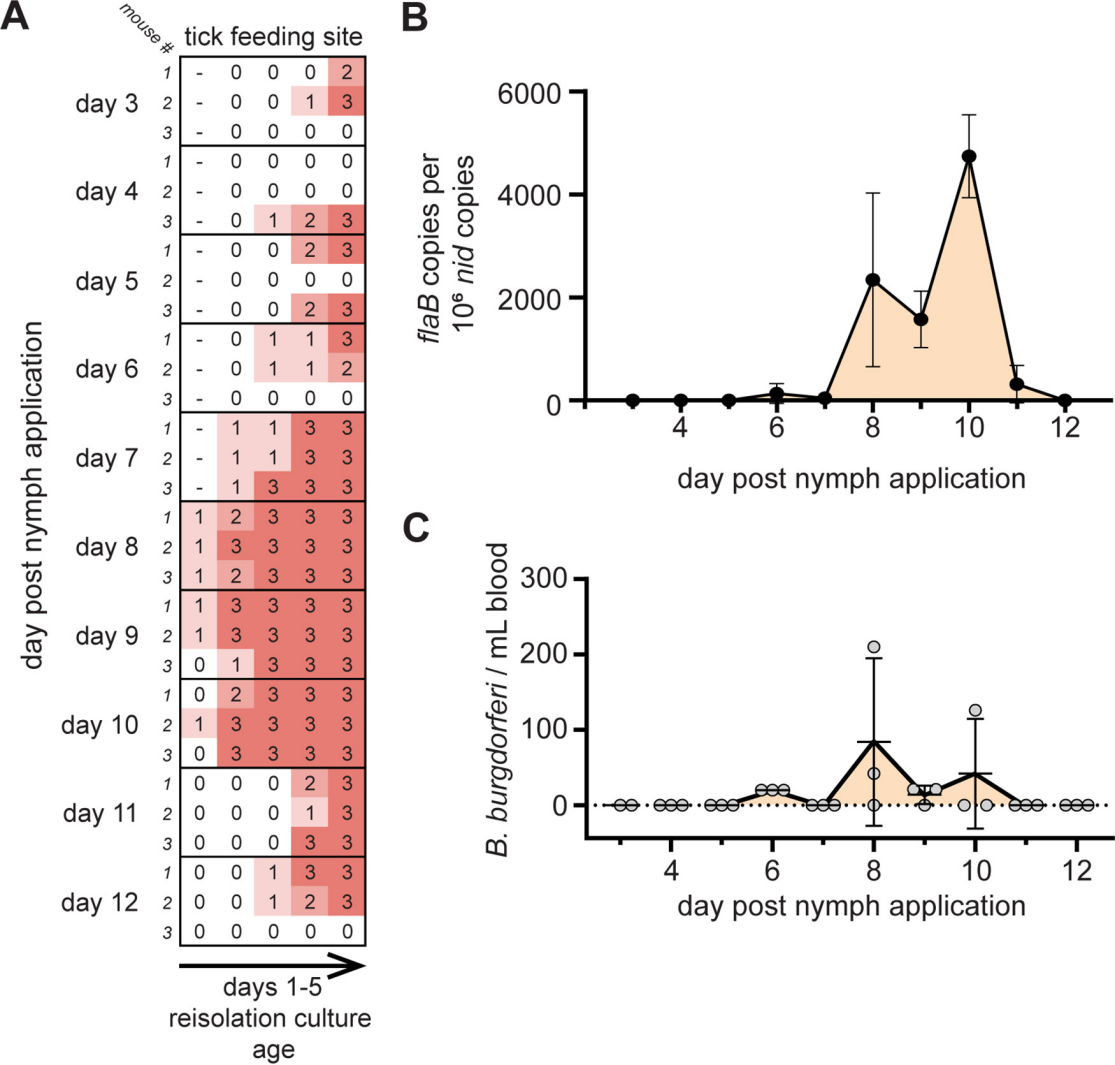
B. burgdorferi spirochetes delivered by tick bite undergo population expansion in mouse skin. Unfed nymphs infected with wild-type (WT) B. burgdorferi were placed in capsules adhered to the dorsal skin of naive C3H/HeN mice. Cohorts of three mice each were assessed for B. burgdorferi infection at the indicated days after nymph application. (A) The tick feeding site was assessed for B. burgdorferi by a tissue reisolation assay. Semiquantitative scoring of reisolation cultures was performed daily over 5 days of culture incubation (0 indicates no spirochetes, and 1 to 3 indicate increasing spirochete densities). (B) Total DNA was extracted from the skin feeding site, and B. burgdorferi loads were measured by quantifying B. burgdorferi *flaB* copies normalized to 10^6^ mouse *nid* copies using quantitative PCR. The mean B. burgdorferi load (black dots) for each cohort was plotted over time. Error bars represent standard deviations. (C) Peripheral blood from each mouse was plated in solid BSK-agarose medium, and circulating B. burgdorferi spirochetes were enumerated by counting CFU. Gray dots represent individual mice. Error bars represent standard deviations.

We previously showed that *bbk13* is important early in B. burgdorferi mammalian infection to promote spirochete expansion in the skin within days after intradermal needle inoculation (13). The deficiency of Δ*bbk13* B. burgdorferi in distal tissue colonization was observed by both needle inoculation (Fig. 1A and B) (13) and natural tick transmission (Fig. 4). We were interested in determining whether wild-type B. burgdorferi undergoes a similar population expansion in the skin after tick bite transmission and if *bbk13* contributes to this process. To achieve this, we confined wild-type B. burgdorferi*-*infected nymphs to capsules affixed to the dorsal skin of mice and determined the kinetics of the B. burgdorferi population in the skin feeding site. The feeding site was harvested daily from cohorts of mice starting at day 3 out to day 12 after nymph application. The spirochete load in the skin was assessed using the semiquantitative reisolation assay as well as quantitative PCR. Feeding sites collected on days 3 to 6 after nymph application showed inconsistent spirochete reisolation, with one or two of the three mice in the cohort showing an absence of spirochetes and peak scores being achieved only at the end of the scoring period in some cultures (Fig. 5A). However, from days 7 to 10 after nymph application, spirochetes were detected in all cultures as soon as day 1 of incubation and reached a peak score of 3 as early as day 2 of incubation (Fig. 5A). Reisolation from skin on days 11 to 12 after nymph application resembled that at the early time points, with spirochetes being detected later in the scoring period (Fig. 5A). Quantification of B. burgdorferi loads from the skin feeding site by quantitative PCR corroborated the findings from the reisolation assay. There were few or no measurable B. burgdorferi spirochetes in the skin feeding site from days 3 to 7 after nymph application, followed by a dramatic increase in the B. burgdorferi load during days 8 to 10 after nymph application (Fig. 5B). A precipitous drop in B. burgdorferi loads was detected on days 11 to 12 (Fig. 5B). Blood was also collected from mice throughout the duration of the nymph capsule feeding experiment and assessed for the presence of disseminating spirochetes in circulation. Plating of whole blood in solid BSK-agarose medium and CFU enumeration revealed peak numbers of B. burgdorferi in the blood on days 8 to 10 after nymph application (Fig. 5C). Taken together, these results show that B. burgdorferi undergoes population expansion in the skin after tick transmission, and the peak of this expansion occurs on days 8 to 10 after nymph application.

### By tick bite transmission, *bbk13* is dispensable for B. burgdorferi early population expansion but critical for disseminated infection.

**FIG 6:**
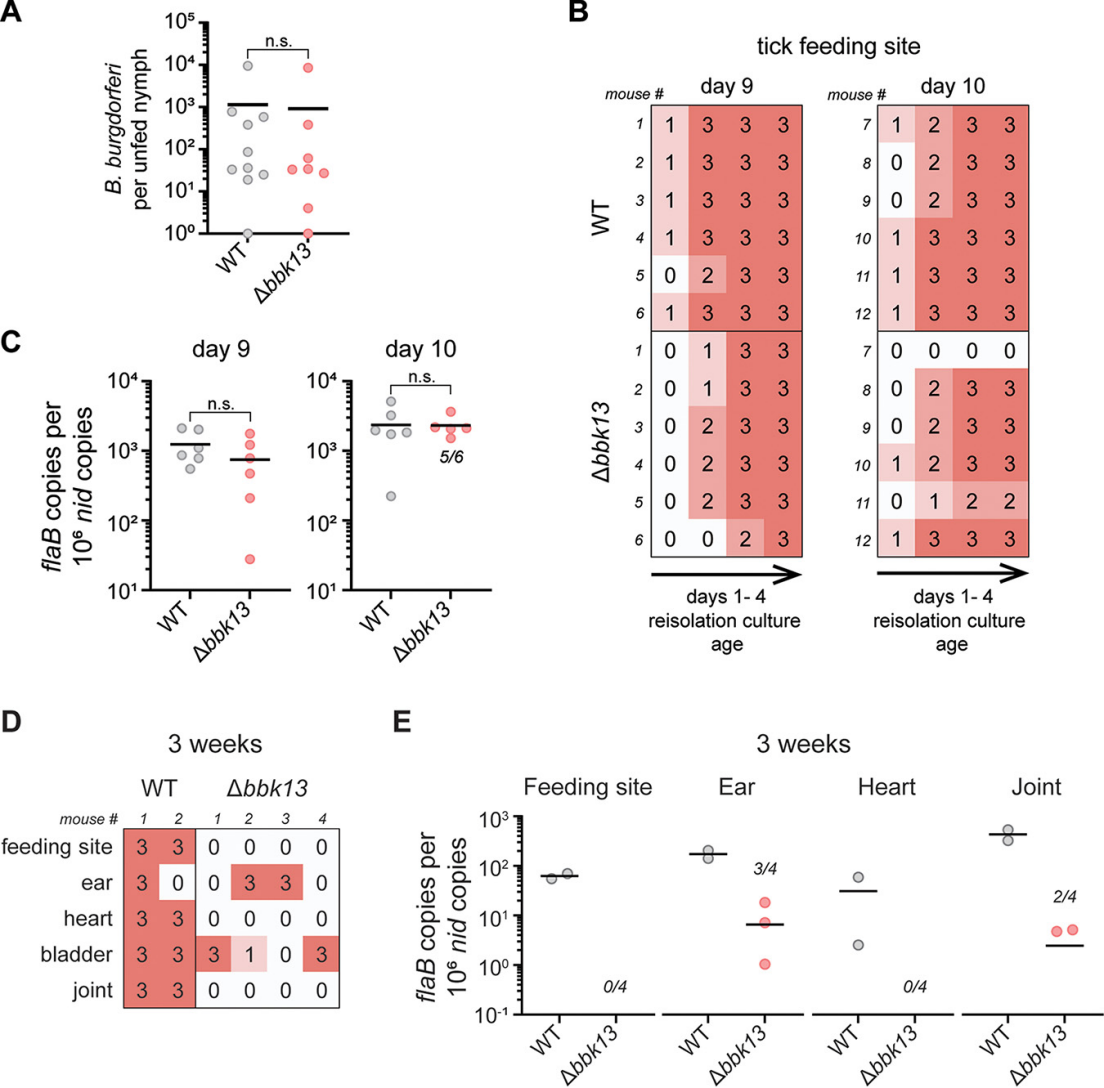
When delivered by tick transmission, *bbk13* is not required for B. burgdorferi population expansion in the skin but is critical for disseminated infection. (A to C) Unfed nymphs infected with wild-type (WT) or Δ*bbk13* B. burgdorferi were placed in capsules adhered to the dorsal skin of groups of six naive C3H/HeN mice to feed. The tick feeding site was collected at days 9 and 10 after nymph application for analysis. (A) Prior to feeding, subsets of individual unfed B. burgdorferi*-*infected nymphs were homogenized and plated in solid BSK-agarose medium to assess spirochete loads by enumerating CFU. (B) The tick feeding site was assessed for B. burgdorferi by a tissue reisolation assay. Semiquantitative scoring of reisolation cultures was performed daily over 4 days of culture incubation (0 indicates no spirochetes, and 1 to 3 indicate increasing spirochete densities). (C) Total DNA was extracted from the tick feeding site. The B. burgdorferi load was measured by quantifying B. burgdorferi *flaB* copies normalized to 10^6^ mouse *nid* copies using quantitative PCR. In cases where not all samples per group had detectable B. burgdorferi DNA, the numbers of positive samples out of 6 mice per group are indicated in the scatterplot. Data points below the level of detection are not shown. The mean is indicated by a horizontal black line. The Mann-Whitney test was used to determine statistical significance (n.s., not significant). (D and E) Unfed nymphs infected with WT or Δ*bbk13* B. burgdorferi were placed in capsules adhered to the dorsal skin of groups of 2 to 4 naive C3H/HeN mice to feed. Three weeks after nymph application, the tick feeding site and various distal tissues were collected for analysis. (D) Tissues were assessed for B. burgdorferi by a tissue reisolation assay. Semiquantitative scoring of reisolation cultures was performed on day 6 of culture incubation (0 indicates no spirochetes, and 1 to 3 indicate increasing spirochete densities). (E) Total DNA was extracted from the indicated tissues. B. burgdorferi loads were measured by quantifying B. burgdorferi *flaB* copies normalized to 10^6^ mouse *nid* copies using quantitative PCR. In cases where not all samples per group have detectable B. burgdorferi DNA, the numbers of positive samples out of the total mice per group are indicated in the scatterplot. Data points below the level of detection are not shown. The mean is indicated by a horizontal black line.

Having established the kinetics of wild-type B. burgdorferi population expansion in the skin following tick transmission, we used the nymph capsule feeding model to assess the contribution of *bbk13* to this process. Unfed nymphs infected with comparable numbers of either wild-type B. burgdorferi or Δ*bbk13* B. burgdorferi spirochetes (Fig. 6A) were placed in feeding capsules on the dorsal skin of C3H/HeN mice to feed to repletion. The skin feeding site was collected on days 9 and 10 after nymph application, which corresponded to the peak of spirochete expansion of tick-transmitted wild-type B. burgdorferi (Fig. 5B). A reisolation assay of the skin site fed on by wild-type B. burgdorferi-infected nymphs collected on both days 9 and 10 demonstrated the detection of spirochetes within 1 day of culture incubation (Fig. 6B). Cultures containing the skin site fed on by Δ*bbk13* B. burgdorferi-infected nymphs showed little reisolation delay compared to wild-type B. burgdorferi, with spirochetes being detected on day 2 of incubation (Fig. 6B). Surprisingly, no difference in B. burgdorferi loads measured by quantitative PCR was detected between wild-type and Δ*bbk13* B. burgdorferi-infected skin sites collected on either day (Fig. 6C). These results are in contrast to previous findings that, when delivered by intradermal needle inoculation, Δ*bbk13* B. burgdorferi failed to undergo efficient population expansion in the skin inoculation site compared to wild-type B. burgdorferi, thus negatively impacting distal tissue colonization at later time points of infection (13). Yet our data indicated that Δ*bbk13* B. burgdorferi is highly attenuated for disseminated mouse infection by free-feeding tick transmission (Fig. 4). Therefore, we examined the disseminated infection outcomes of Δ*bbk13* B. burgdorferi delivered by nymph capsule feeding. Three weeks after nymph application in feeding capsules, the feeding site and multiple distal tissues were dissected and assessed for spirochete colonization. Interestingly, despite the lack of B. burgdorferi load differences between wild-type and Δ*bbk13* B. burgdorferi at the feeding site early in infection, Δ*bbk13* B. burgdorferi demonstrated reduced numbers in all tissues examined at this later time point. Nearly all reisolation cultures containing tissues from mice fed on by wild-type B. burgdorferi-infected nymphs were positive for spirochetes, while only 5 out of 20 cultures were positive for spirochetes from mice fed on by Δ*bbk13* B. burgdorferi-infected nymphs (Fig. 6D). Furthermore, quantitative PCR analysis of spirochete loads consistently showed reduced or undetectable Δ*bbk13* B. burgdorferi spirochetes in tissues (Fig. 6E), indicating the critical requirement for *bbk13* for productive disseminated infection.

### Δ*bbk13* B. burgdorferi infectivity is restored in immunocompromised mice.

**FIG 7:**
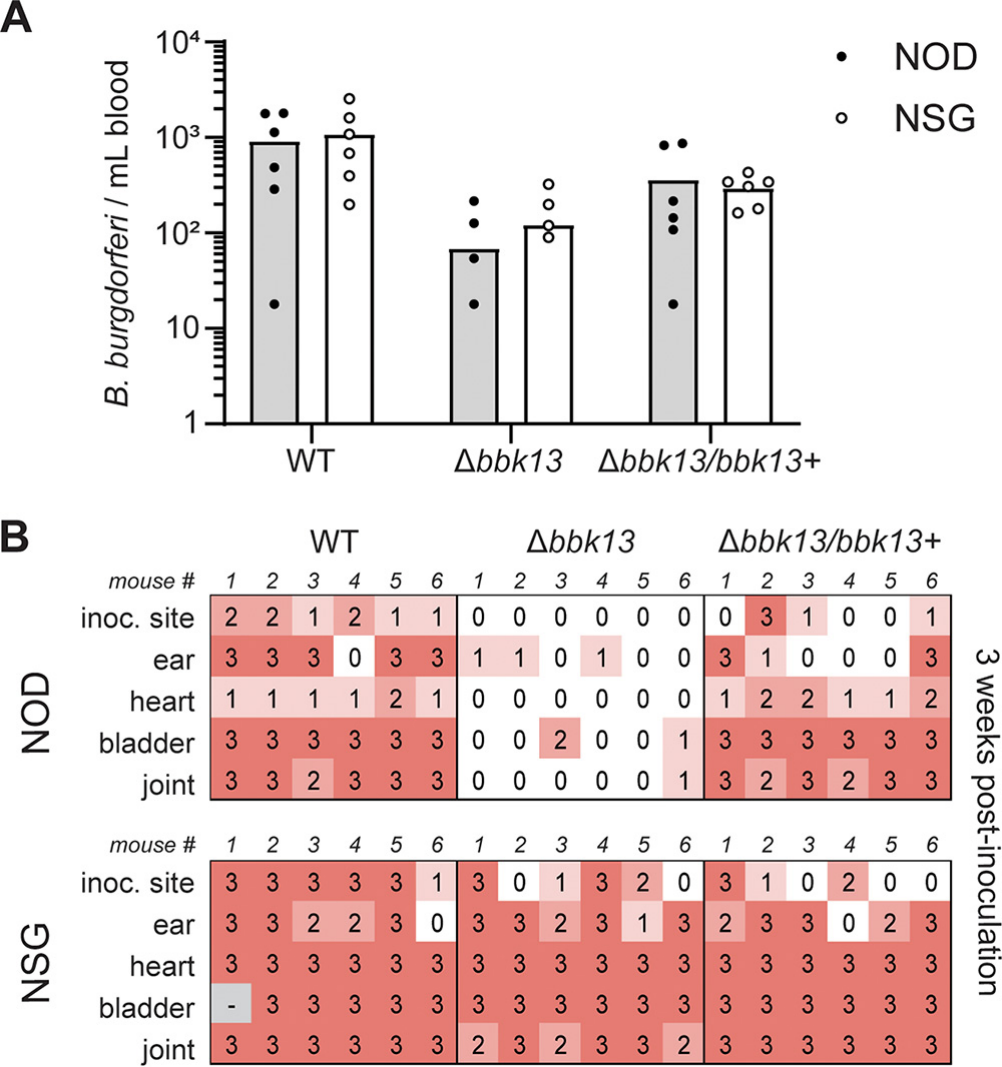
Infectivity of Δ*bbk13* B. burgdorferi is restored in immunocompromised mice. Groups of six control NOD/ShiLtJ (NOD) or immunocompromised Nod-*scid* IL2Rγ^null^ (NSG) mice were intradermally inoculated with 10^4^ wild-type (WT), Δ*bbk13*, or Δ*bbk13*/*bbk13*^+^ B. burgdorferi spirochetes. (A) At 6 days postinoculation, blood was collected and assessed for disseminating spirochetes by plating in solid BSK-agarose medium and enumerating CFU. (B) At 3 weeks postinoculation, tissues were assessed for B. burgdorferi by a reisolation assay. Semiquantitative scoring of reisolation cultures was performed on day 5 of culture incubation (0 indicates no spirochetes, and 1 to 3 indicate increasing spirochete densities).

The above-described results show that delivery by tick bite locally rescued the defect of Δ*bbk13* B. burgdorferi in spirochete expansion in the skin during early infection. It is known that the feeding activity of *Ixodes* ticks leads to immunomodulation at the bite site, promoting B. burgdorferi survival (9, 17), which therefore prompted the question as to whether *bbk13* is involved in immune evasion. To test this, we determined whether Δ*bbk13* B. burgdorferi mammalian infectivity was restored if the host immune response was reduced overall. We delivered 10^4^ wild-type, Δ*bbk13*, or Δ*bbk13*/*bbk13*^+^ B. burgdorferi spirochetes by intradermal needle inoculation into highly immunocompromised Nod-*scid* IL2Rγ^null^ (NSG) mice and the congenic control strain, Nod/ShiLtJ. At day 6 postinoculation, blood was collected from mice and plated in solid BSK-agarose medium to enumerate circulating B. burgdorferi spirochetes. No B. burgdorferi clone-specific differences were observed between the number of circulating B. burgdorferi spirochetes in immunocompromised NSG mice and that in the control mouse strain (Fig. 7A). At 3 weeks postinoculation, infection of the inoculation site and distal tissues was assessed by a semiquantitative reisolation assay. As expected, Δ*bbk13* B. burgdorferi spirochetes were highly attenuated for infection of the control mice, with only 6 out of 30 cultures having a score of 1 or 2 following 5 days of incubation. In contrast, 29 out of 30 and 24 out of 30 reisolation cultures from wild-type B. burgdorferi- and Δ*bbk13*/*bbk13*^+^ B. burgdorferi-infected NOD mice, respectively, received a score of 1 to 3 at the same time point (Fig. 7B, top). Strikingly, in immunocompromised NSG mice, Δ*bbk13* B. burgdorferi demonstrated robust infection. Twenty-one out of 30 reisolation cultures from Δ*bbk13* B. burgdorferi-inoculated NSG mice received a score of 3 following 5 days of incubation, congruent with those of wild-type B. burgdorferi (25/30 cultures) and Δ*bbk13*/*bbk13*^+^ B. burgdorferi (22/30 cultures) (Fig. 7B, bottom). These data indicated that without host immune pressure, the infection defect of Δ*bbk13* B. burgdorferi was overcome, resulting in productive disseminated infection of distal tissues and suggesting a role for BBK13 in immune evasion.

## DISCUSSION

The maintenance of B. burgdorferi in the natural reservoir depends on its ability to colonize a vertebrate host, to position itself to be acquired during tick feeding, and to subsequently be transmitted to a new vertebrate host. Although B. burgdorferi colonizes several mammalian tissues that contribute to the pathogenesis of Lyme disease in humans, colonization of the skin of reservoir hosts is most pertinent to the upkeep of the enzootic cycle. Intravital imaging of the skin of infected mice revealed that B. burgdorferi undergoes directed migration toward a feeding tick (18). Furthermore, B. burgdorferi has shown positive chemotactic responses to components of the tick salivary gland and that these factors are important during spirochete acquisition by feeding ticks (19, 20). This evidence supports the ability of B. burgdorferi to sense the presence of the feeding tick and to position itself to best be taken up by a feeding tick. However, these cues are likely restricted to the immediate vicinity of an active tick feeding site. Therefore, efficient B. burgdorferi acquisition likely depends on achieving the appropriate density of B. burgdorferi spirochetes in the skin such that enough B. burgdorferi spirochetes will encounter the cues from a feeding tick. B. burgdorferi spirochetes must then be present in the feeding site at a sufficient number for successful tick acquisition and continuation of the enzootic cycle.

We and others have previously described the population expansion of B. burgdorferi at the site of needle inoculation (13, 21–24). We further showed that this population expansion is promoted by gene *bbk13*, and the inability to expand in numbers at the skin inoculation site leads to attenuated dissemination and distal tissue colonization (13). We now show that naive larvae fail to acquire Δ*bbk13* B. burgdorferi from needle-inoculated mice. Our data suggest that the number of Δ*bbk13* B. burgdorferi spirochetes colonizing the skin is likely below the threshold required for successful tick acquisition but do not rule out the possibility of a specific function of *bbk13* as a driver of the acquisition defect. Matching the wild-type and Δ*bbk13* B. burgdorferi numbers in the skin would allow any *bbk13*-specific requirement to be tested during tick acquisition. However, increasing the needle inoculum dose of the mutant did not result in increased spirochete loads in the skin.

Using the semiquantitative tissue reisolation assay as a measure of tissue burden and highly immunocompromised NSG mice, we discovered that when host immune pressures are relieved, Δ*bbk13* B. burgdorferi is able to colonize distal tissues similarly to wild-type B. burgdorferi. Thus, it appears that *bbk13* is important for circumventing the host immune response, either directly or indirectly, to promote B. burgdorferi survival in the mammalian host. The restoration of Δ*bbk13* B. burgdorferi loads in the tissues of NSG mice to apparent wild-type levels suggests that this model could be used to test for a *bbk13*-specific requirement for tick acquisition. However, there are a number of limitations to this model in its ability to address this question. The semiquantitative nature of the tissue reisolation assay does not provide sufficient resolution at a score of 3 (>15 spirochetes in the field of view) to precisely distinguish quantitative differences in tissue loads between wild-type and Δ*bbk13* B. burgdorferi infections. Furthermore, assessment of spirochete loads in distal tissues of NSG mice using quantitative PCR enumeration of B. burgdorferi genome copies was unreliable. Tissues that had no reisolation of live spirochetes recorded high spirochete loads by qPCR (data not shown). This observation indicates that B. burgdorferi DNA is retained in the tissues despite the absence of viable spirochetes. The NSG mice and the congenic control strain used in our studies are of the Nod/ShiLtJ background, commonly called NOD (nonobese diabetic). NOD mice have been shown to have poor clearance of apoptotic cells and the generation of autoantibodies against DNA (25), which indicates poor clearance of DNA. Moreover, hyperglycemic mice are suspected to suffer from deficiencies in clearing B. burgdorferi debris and DNA from tissues (26). This is an important consideration for mouse infection studies since quantitative PCR measurement of B. burgdorferi genome copies in distal tissues, the current gold standard for assessing B. burgdorferi tissue loads, assumes a correlation between the amounts of B. burgdorferi genomic DNA and viable spirochetes. The caveats of precise quantitation of B. burgdorferi in the tissues of NSG mice have the potential to confound the interpretation of tick acquisition, or the lack thereof, of Δ*bbk13* B. burgdorferi. Therefore, NSG mice were deemed not to be the appropriate model to test this question. In addition, while the removal of immune pressures clearly allowed Δ*bbk13* B. burgdorferi to achieve disseminated infection, given the technical limitations of these mice, we cannot rule out the potential that BBK13 also contributes to a broader mechanism of dissemination not overcome by the immunodeficiency of the NSG background. Nonetheless, the broadly immunocompromised background of NSG mice is a useful tool to eliminate a large swath of the host immune response across both innate and adaptive arms. Future studies will focus on the identification of the specific aspect(s) of the host immune response that BBK13 contributes to providing protection against.

Consistent with previous findings for B. burgdorferi lacking the entire linear plasmid 36 (lp36) (27), on which the *bbk13* gene resides (28), no *bbk13*-dependent defect in B. burgdorferi replication or survival was detected across the tick life stages. Tick bite transmission of B. burgdorferi induces significant changes in both the spirochete and the mammalian host to promote infection (8, 9), which are not recapitulated by *in vitro*-grown B. burgdorferi delivered by needle inoculation. Therefore, we sought to assess the contribution of the *bbk13* gene to mouse infection by tick bite transmission. B. burgdorferi spirochetes lacking *bbk13* were highly attenuated for disseminated infection 3 weeks following free feeding of infected ticks on mice. Our previous findings indicated a role for *bbk13* in B. burgdorferi population expansion in the skin site of infection and the importance of this population expansion for the establishment of productive disseminated infection (13). To investigate the contribution of *bbk13* to this model of infection in the context of tick bite transmission, we first established the early kinetics of wild-type B. burgdorferi in the skin site of tick feeding using capsule-confined nymphs. Similar to the spirochete numbers in the skin delivered by needle inoculation, tick-delivered B. burgdorferi underwent population expansion in the feeding site, with peak numbers of spirochetes being detected on days 8 to 10 after nymph application. Given that B. burgdorferi transmission can occur 24 to 48 h after the start of tick feeding and that tick feeding is not synchronized, the observed kinetics of spirochete population expansion in the skin was comparable to that determined by needle inoculation (13). Using the nymph capsule feeding approach, analysis of the numbers of Δ*bbk13* B. burgdorferi spirochetes in the tick feeding site on days 9 and 10 after nymph application revealed the striking result that the loss of *bbk13* had no impact on spirochete population expansion in the skin. It is important to note that peak numbers of wild-type B. burgdorferi spirochetes in the blood were also detected on days 8 to 10 after nymph application. As the skin is a highly vascularized tissue and the mice were not perfused prior to harvest of the tick feeding site, it remains a possibility that circulating B. burgdorferi spirochetes in the blood may have contributed to the numbers of wild-type and Δ*bbk13* B. burgdorferi spirochetes detected in the skin at these time points. Despite the amelioration of the early infection defect of Δ*bbk13* B. burgdorferi when delivered by tick feeding, assessment of mice fed on by capsule-confined nymphs 3 weeks after nymph application revealed that the *bbk13* gene remained critical for disseminated infection. Together, these data suggest that one or more of the biological changes that the spirochetes and/or the host undergoes during tick feeding allowed Δ*bbk13* B. burgdorferi to overcome the barrier to population expansion in the skin. However, the relief of this early phenotype was not sufficient to overcome the need for *bbk13* during disseminated infection. These findings emphasize that B. burgdorferi population expansion in the skin and dissemination to distal tissues are distinct events of mammalian infection that are uniquely influenced by the feeding tick and likely require different sets of B. burgdorferi genes. Furthermore, this work underscores the significance of the vector as a driver of vector-borne pathogen adaptation and pathogenesis.

Our studies have provided greater insight into the role of *bbk13* in the biology of B. burgdorferi, yet the molecular function of BBK13 remains unknown. Recently, we established that BBK13 forms large oligomeric complexes in the spirochete membrane (14). Current work is focused on investigating how BBK13 oligomerization contributes to the mechanisms by which B. burgdorferi surmounts immune barriers in the mammalian host in order to establish a disseminated infection. In sum, this work cements the importance of *bbk13* in promoting B. burgdorferi mammalian infectivity and highlights the influence of the tick vector on the pattern of infection, particularly at the skin—the interface between host, vector, and pathogen.

## MATERIALS AND METHODS

### B. burgdorferi clones and growth conditions.

The *Borrelia* (*Borreliella*) *burgdorferi* clones used in this study were derived from the low-passage-number infectious clone B31 A3-68 Δ*bbe02*, referred to here as the wild type, which lacks plasmids cp9 and lp56 as well as the *bbe02* gene on lp25 (29). The Δ*bbk13* mutant clone and the Δ*bbk13/bbk13*^+^ complemented strain have been described previously (13). B. burgdorferi cultures were grown at 35°C in liquid Barbour-Stoenner-Kelly II (BSKII) medium containing gelatin and 6% rabbit serum. Alternatively, B. burgdorferi spirochetes were plated in solid BSK-agarose medium and incubated at 35°C under 2.5% CO_2_. The following antibiotics were used, as needed: kanamycin (200 μg/ml), streptomycin (50 μg/ml), and gentamicin (40 μg/ml).

### Ethics statement.

The University of Central Florida is accredited by the International Association for Assessment and Accreditation of Laboratory Animal Care. Protocols for all animal experiments were prepared according to the guidelines of the National Institutes of Health and were reviewed and approved by the University of Central Florida Institutional Animal Care and Use Committee.

### Needle inoculation of mice.

B. burgdorferi cultures were grown from frozen glycerol stocks in BSKII medium with the appropriate antibiotics to stationary phase (1 × 10^8^ spirochetes/ml). B. burgdorferi cultures were kept at stationary phase for ∼24 h prior to mouse inoculation. Culture density was determined using a Petroff-Hauser chamber under dark-field microscopy. B. burgdorferi cultures were diluted in BSKII medium to the desired inoculum dose. Groups of C3H/HeN, Nod-*scid* IL2Rγ^null^ (NSG), or NOD/ShiLtJ (NOD) mice were inoculated intradermally into the shaved dorsal skin with wild-type, Δ*bbk13*, or Δ*bbk13/bbk13*^+^ B. burgdorferi at a dose of 10^4^, 10^5^, 10^6^, or 10^7^ spirochetes. All inoculum cultures were analyzed for plasmid content by PCR and plated in solid BSK-agarose medium to verify the presence of virulence plasmids lp25, lp28-1, and lp36 in individual colonies (27). All inoculum cultures had the expected plasmid profile, and 80% to 100% of the individual clones examined contain all three virulence plasmids. Seven days or 3 to 4 weeks after tick feeding, mice were assayed for B. burgdorferi infection by a semiquantitative tissue reisolation assay (as described below) (13), quantitative PCR loads in tissues (13, 27), and/or serology (30).

### B. burgdorferi acquisition by naive *Ixodes* larvae.

Three weeks after inoculation with wild-type, Δ*bbk13*, or Δ*bbk13/bbk13*^+^ B. burgdorferi, mice were fed upon by groups of ∼150 naive Ixodes scapularis larval ticks (CDC, BEI Resources, or Oklahoma State University). Replete larvae were collected and individually assessed for B. burgdorferi infection, as described below.

### Artificial infection of *Ixodes* larvae and free-feeding tick transmission.

Approximately 4-month-old naive Ixodes scapularis larval ticks (CDC, BEI Resources, or Oklahoma State University) were dehydrated by exposure to saturated ammonium sulfate for 24 h. Log-phase-grown B. burgdorferi clones were diluted to 2 × 10^7^ cells/ml in BSKII medium. Five hundred microliters of the spirochetes was incubated with dehydrated ticks at 35°C for ∼1.5 h and washed twice with phosphate-buffered saline (PBS) (15). The inoculum cultures were verified to contain the expected endogenous plasmids (27, 31). Infectious plasmids lp25, lp28-1, and lp36 were present in 80 to 100% of individuals of each inoculum culture. Three days after artificial infection, cohorts of ∼150 larvae were fed to repletion on groups of 5 naive C3H/HeN mice (Envigo) per B. burgdorferi clone. A subset of fed larvae was allowed to molt into nymphs, and subsequently, cohorts of 25 nymphs were fed on groups of 5 naive C3H/HeN mice (Envigo) per B. burgdorferi clone. Three weeks after tick feeding, mice were assayed for B. burgdorferi infection by a semiquantitative tissue reisolation assay (as described below) (13), quantitative PCR loads in tissues (13, 27), and/or serology (30).

### Capsule feeding tick transmission.

The top portion of a 2-ml screw-cap tube with a perforated cap (capsules) was glued onto the shaved dorsal skin of groups of 2 to 6 C3H/HeN mice using veterinary tag cement (Nasco) and allowed to completely adhere overnight. Groups of 15 B. burgdorferi-infected unfed nymphs were added to each capsule and allowed to feed. Seven days after nymph application, replete nymphs were collected. Seven days or 3 weeks after nymph application, mice were euthanized, and the skin feeding site and distal tissues were collected for analysis. Infection was determined by a semiquantitative tissue reisolation assay (as described below) (13), quantitative PCR loads in tissues (13, 27), and/or serology (30).

### Semiquantitative tissue reisolation scoring method.

Semiquantitative tissue reisolation scoring was performed as described previously (13). Briefly, tissues were dissected at the indicated time points after inoculation and placed into BSKII medium containing an antibiotic cocktail of rifampin (50 μg/ml), amphotericin B (2.5 μg/ml), and phosphomycin (20 μg/ml) (RPA cocktail). B. burgdorferi is naturally resistant to these antibiotics. Reisolation cultures were incubated at 35°C. Cultures were visually inspected daily for the presence of spirochetes using dark-field microscopy. While viewing at a ×200 magnification, a numerical score is assigned, as follows: 0 for the absence of spirochetes, 1 for a low spirochete density (<5 spirochetes in the field of view), 2 for a medium spirochete density (5 to 15 spirochetes in the field of view), and 3 for a high spirochete density (>15 spirochetes in the field of view). Finally, the presence or absence of spirochetes (denoted by “y” [yes] or “n” [no], respectively) was scored at the endpoint of the assay, after 14 days of incubation.

### Quantification of B. burgdorferi loads in *Ixodes* ticks.

The B. burgdorferi densities in ticks were assessed before and 7 to 14 days after feeding to repletion on mice. Ticks were surface sterilized using sequential washes with 3% hydrogen peroxide, 70% ethanol, and sterile water. Groups of 10 unfed larvae, individual fed larvae, or individual nymphs (fed or unfed) were crushed and homogenized in BSKII medium using a plastic pestle in microcentrifuge tubes, first by hand and then by motorized adapter. Homogenates were serially diluted and plated in solid BSK-agarose medium supplemented with the RPA cocktail. CFU were enumerated, and the B. burgdorferi load per tick was calculated (30, 32). Alternatively, total DNA was isolated from groups of 40 unfed larvae using a NucleoSpin tissue kit (Clontech Laboratories) according to the manufacturer’s specifications, and qPCR was performed as previously described (30).

### Quantification of B. burgdorferi density in the blood.

Peripheral blood was collected from mice on day 6 after needle inoculation with B. burgdorferi or days 3 to 12 after application of B. burgdorferi-infected ticks. Whole blood was serially diluted in BSKII medium, plated in solid BSK-agarose medium plus the RPA cocktail, and incubated at 35°C under 2.5% CO_2_. CFU were enumerated, and the spirochete density in circulating blood was calculated (13).

### Graphs, figures, and statistical tests.

GraphPad Prism v.9 was used to generate graphs and perform statistical tests. Adobe Illustrator was used for figure assembly and minor editing of graphic elements.
